# Lutetium-177 PSMA radioligand therapy in taxan-naive first- and second-line metastatic castration resistant prostate cancer after first-line ARPI therapy

**DOI:** 10.1007/s00259-025-07076-7

**Published:** 2025-01-13

**Authors:** Mike Wenzel, Benedikt Hoeh, Carolin Siech, Florestan Koll, Clara Humke, Daniel Groener, Thomas Steuber, Markus Graefen, Tobias Maurer, Severine Banek, Felix K. H. Chun, Philipp Mandel

**Affiliations:** 1 Department of Urology, University Hospital Frankfurt, Goethe University Frankfurt Am Main, Frankfurt, Germany; 2 Department of Nuclear Medicine, University Hospital Frankfurt, Goethe University Frankfurt Am Main, Frankfurt, Germany; 3 Martini-Klinik Prostate Cancer Center, University Hospital Hamburg-Eppendorf, Hamburg, Germany

**Keywords:** MCRPC, PFS, OS, Survival, Lu-177

## Abstract

**Purpose:**

Lutetium-177 Prostate-specific membrane antigen (Lu-PSMA) radioligand therapy is EMA-approved for metastatic castration resistant prostate cancer (mCRPC) after androgen receptor pathway inhibition (ARPI) and taxan-based chemotherapy. However, its effect in taxan-naïve patients is under current investigation.

**Methods:**

We relied on the FRAMCAP database to elaborate Lu-PSMA therapy outcomes of progression-free (PFS) and overall (OS) in taxan-naïve mCRPC patients after previous ARPI treatment. Comparison was made against current standard of care with ARPI or docetaxel, irrespective of the previous used staging modality.

**Results:**

Of 269 patients, 11% received Lu-PSMA in first/second-line mCRPC vs. 57% ARPI vs. 33% docetaxel. Mostly no significant baseline differences between Lu-PSMA and ARPI patients were observed, while Lu-PSMA patients were significantly older, received less systematic treatments and ECOG1-2 proportions were higher, relative to docetaxel patients. In PFS (13.3 vs. 8.2 months, hazard ratio [HR]: 0.70, *p* = 0.16) and OS analyses (68.9 vs. 39.1 months, HR: 0.64, *p* = 0.2), Lu-PSMA was numerically more favorable than ARPI. In additional multivariable Cox regression models, Lu-PSMA was significant better regarding PFS and OS, relative to ARPI (both *p* < 0.05). Compared to docetaxel, also significant better PFS (13.3 vs. 8.1 months, HR: 0.46) and OS (68.9 vs. 27.3 months, HR: 0.34, both *p* < 0.01) was observed for Lu-PSMA treatment. The OS advantage was also observed after multivariable adjustment (*p* < 0.01).

**Conclusion:**

This retrospective single-center study including a substantial proportion of patients with treatment preference for Lu-PSMA suggests that Lu-PSMA therapy provides significantly more favorable PFS and OS outcomes in taxan-naïve mCRPC patients after previous ARPI treatment, relative to ARPI or docetaxel treatment and may be considered as an early mCRPC treatment option.

**Supplementary Information:**

The online version contains supplementary material available at 10.1007/s00259-025-07076-7.

## Introduction

Patients with metastatic castration resistant prostate cancer (mCRPC) are at high risk of cancer-specific death and severe disease-related side effects due to tumor burden. However, in mCRPC several different treatment options are currently approved as life-prolonging agents [1–6]. Still, optimal sequencing of mCRPC therapies are under current scientific investigation, since progression-free survival (PFS) rates decrease within every newly administered advanced treatment line [7–9].

Recently in 2022, the European Medical Agency (EMA) approved Lutetium-vipivotidtetraxetan prostate-specific membrane antigen radioligand therapy (Lu-PSMA) for mCRPC patients after previous treatment with androgen receptor pathway inhibitors (ARPI) and taxan-based chemotherapy, based on the results of the Vision trial [10]. With its cancer-control effects and a different approach relative to ARPI or taxan-based chemotherapy and as a treatment delivering beta radiation to a molecular target specifically adherent to prostate cancer cells, Lu-PSMA therapy became a crucial part within the sequential therapies of mCRPC patients. Therefore, the optimal time point of Lu-PSMA therapy administration is especially under scientific investigation. So far, the PSMAfore study and the preliminary study result presented at the European Society Medical Oncology (ESMO) congress 2024 of the SPLASH study and one previously published trial result (ENZA-p) suggested beneficial PFS outcomes with Lu-PSMA therapy vs. ARPI or its combination also in earlier stages of first line or taxan-naïve mCRPC patients [11–13]. Moreover, currently only one real-world study addressed this important topic without comparing Lu-PSMA vs. current standard of care of ARPI or chemotherapy [14].

We addressed this void and relied on the FRAMCAP database (Frankfurt Metastatic Cancer Database of the Prostate) to compare cancer-control outcomes such as PFS and overall survival in Lu-PSMA treated taxan-naïve mCRPC patients vs. the current standard of care. We hypothesized that in a real-world scenario important differences in cancer-control outcomes may exist regarding taxan-naïve mCRPC patients treated with Lu-PSMA vs. ARPI or docetaxel.

## Materials and methods

### Study population

After obtaining approval from the local ethics committee (reference number: SUG-5–2024) and adhering to the principles of the Declaration of Helsinki, we conducted a retrospective review of all mCRPC patients in the prospectively collected FRAMCAP database. The FRAMCAP with 1,180 metastatic prostate cancer patients treated at the Department of Urology and discussed within a multimodal tumor board of the University Hospital Frankfurt, Germany since year 2014, were screened. For analyses, only taxan-naïve prostate cancer patients who had received either at least one cycle of Lu-PSMA, docetaxel or another line of ARPI and had one prior ARPI treatment for metastatic hormone-sensitive prostate cancer (mHSPC) or mCRPC were included. This inclusion criteria yielded 269 mCRPC patients for analyses. Staging was mainly done by conventional imaging (CT-scan and bone scan), while in patients receiving Lu-PSMA a PSMA-PET/CT scan with PSMA-positive lesions was mandatory.

### Lu-PSMA tracer and competitors

Treatment of Lu-PSMA was administered at the nuclear medicine department every 4–6 weeks, as previously described and further outlined in the supplements [15]. Lu-PSMA could be administered for patients as an individual compassionate use after previous case discussion in a multimodal tumor board.

As a competitor for Lu-PSMA analyses, ARPI or docetaxel treatment was used. mHSPC/mCRPC ARPI treatment was either apalutamide (240mg/day, only mHSPC), abiraterone (1000mg/day), enzalutamide (160mg/day). Docetaxel chemotherapy was administered three-weekly as a dose of 25mg/m^2 up to ten cycles. Adoptions in treatments, dosage or intervals were made according to patients’ individual response and side effects. Treatment with Lu-PSMA, ARPI or docetaxel was stopped after suspicion of progression (clinically or biochemically) validated via radiographic re-staging or patients’ withdrawn.

### Statistical analysis

Descriptive statistics involved calculating the frequencies and proportions of the categorical variables used in the analyses. For continuous variables, median values and interquartile ranges (IQR) were reported. The Chi-square test was used to determine the statistical significance of differences in proportions, while the t-test and Kruskal-Wallis test were used to analyze differences in distributions.

In the first step of analyses, PFS and OS outcomes were analyzed by comparing Lu-PSMA treatment vs. ARPI. In the second step of the analyses, all cancer-control outcome measurements were repeated for Lu-PSMA treatment vs. docetaxel chemotherapy. Progression was defined as the beginning of another systemic treatment.

For all cancer-control outcome estimates, univariable, as well as multivariable Cox regression models were applied. Adjustment in multivariable Cox regression models were performed for metastatic sites at mCRPC (M1a vs. M1b vs. M1c), age at mCRPC, Gleason Score, Eastern Cooperative Oncology Group (ECOG) status at metastatic disease, year of treatment and additionally for OS analyses for the number of treatment lines received. All tests were two sided with a level of significance set at *p* < 0.05. R software environment for statistical computing and graphics (version 3.4.3) was used for all analyses.

## Results

Of overall 269 mCRPC patients (Table 1), median age at mCRPC was 72 years (IQR: 67–79 years) with a median PSA at mCRPC of 17 ng/ml (IQR: 6–47 ng/ml). In total, 54% of all included patients were categorized as ECOG status 1–2 and 37% harbored any active or treated of cardiovascular disease. De Novo metastatic were initially 54% of patients and 42% received any type of local treatment to the prostate. Median number of received systemic treatments for mCRPC were three (IQR: 2–4). Median PSA response in the mCRPC line of interest was 23% (IQR: 4–62%). At metastatic hormone-sensitive prostate cancer (mHSPC), 53% of patients harbored high volume metastatic burden. At mCRPC, the majority of patients harbored bone metastases only (83%), while 12% vs. 5.5% harbored lymph node or visceral (± bone) metastases. Overall, 36% of included patients received treatment of interest within the first-line mCRPC setting.

**Table 1:** Characteristics of 269 metastatic castration resistant prostate cancer (mCRPC) patients stratified according to treatment in first- or second-line mCRPC with 177-lutetium prostate-specific membrane antigen (Lu-PSMA) vs. androgen receptor pathway inhibitor (ARPI) or Lu-PSMA vs. docetaxel chemotherapy

			A			B	
Characteristic	**N**	Overall *N* = 269^*1*^	Lu-PSMA, *N* = 29 (11%)^a^	ARPI, *N* = 152 (57%)^a^	*p*-value^b^	Docetaxel, *N* = 88 (33%)^a^	*p*-value^b^
Age at metastatic disease, years	256	71 (65, 77)	74 (69, 79)	72 (66, 77)	0.055	68 (62, 73)	< 0.001
Age at mCRPC, years	167	72 (67, 79)	76 (73, 83)	74 (68, 79)	0.083	69 (64, 75)	< 0.001
PSA at mCRPC, ng/ml	133	17 (6, 47)	23 (9, 82)	18 (5, 47)	0.2	12 (4, 40)	0.091
Systemic treatment lines for mCRPC	269	3 (2, 4)	2 (1, 2)	3 (2, 4)	< 0.001	3 (3, 5)	< 0.001
Received cycles	139	3 (2, 6)	3 (2, 5)			4 (2, 6)	0.2
PSA response, %	26	23 (4, 62)	20 (11, 30)	17 (0, 90)	0.9	41 (17, 62)	0.3
ECOG at mCRPC	87				0.13		0.017
0		40 (46%)	4 (24%)	17 (45%)		19 (59%)	
1–2		47 (54%)	13 (76%)	21 (55%)		13 (41%)	
Cardiovascular disease	171	64 (37%)	9 (50%)	33 (34%)	0.2	22 (39%)	0.4
Gleason score 8–10	234	165 (71%)	16 (62%)	89 (69%)	0.5	60 (76%)	0.2
Local therapy	269	112 (42%)	12 (41%)	52 (34%)	0.5	48 (55%)	0.2
De Novo metastatic disease	259	140 (54%)	15 (54%)	86 (59%)	0.6	39 (45%)	0.4
High volume mHSPC	104	55 (53%)	6 (67%)	35 (54%)	0.7	14 (47%)	0.5
Metastatic sites at mCRPC	110				0.8		0.4
M1a		13 (12%)	2 (11%)	5 (10%)		6 (15%)	
M1b		91 (83%)	15 (79%)	42 (84%)		34 (83%)	
M1c		6 (5.5%)	2 (11%)	3 (6.0%)		1 (2.4%)	
Pre-treatment	269						
ARPI		269 (100%)	29 (100%)	152 (100%)		88 (100%)	
Docetaxel		0 (0%)	0 (0%)	0 (0%)		0 (0%)	

Abbreviations: *PSA,* Prostate-specific antigen; *ECOG,* Eastern Cooperative Oncology group; *mHSPC,* metastatic hormone-sensitive prostate cancer; *ADT,* Androgen deprivation therapy; *nmHSPC,* non-metastatic hormone-sensitive prostate cancer; *m0CRPC,* non-metastatic CRPC

^a^Median (Q1, Q3); n (%);^*2*^ Kruskal-Wallis rank sum test; Fisher’s exact test; Pearson’s Chi-square test

^b^Kruskal-Wallis rank sum test; Fisher’s exact test; Pearson’s Chi-square test

### Baseline characteristics: Lu-PSMA vs. ARPI

Of all included 269 patients, 11% (*n* = 29) received Lu-PSMA therapy in first- or second-line mCRPC vs. 57% (*n* = 152) ARPI treatment. In comparison between both treatments (Table 1A), patients received more systemic treatments for mCRPC in median than Lu-PSMA (2 vs. 3, *p* < 0.001). Despite not reaching significance, ECOG status 1–2 was more frequent in Lu-PSMA patients (76% vs. 55%, *p* = 0.1), similar to cardiovascular diseases (50% vs. 34%, *p* = 0.2), relative to ARPI patients. Patients with Lu-PSMA had almost double the number of M1c disease at mCRPC (11 vs. 6%). No further clinically meaningful or statistically significant differences were observed in comparison regarding De Novo metastatic disease, local therapies to the prostate.

### PFS and OS analyses: Lu-PSMA vs. ARPI

**Fig. 1:**
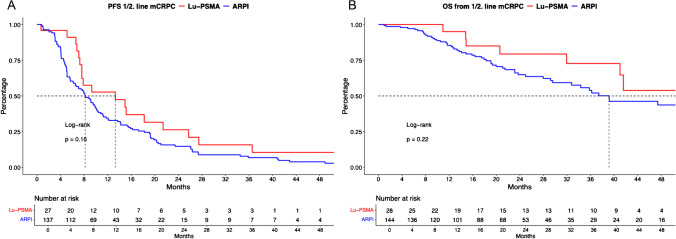
Kaplan Meier curves depicting progression-free survival (PFS, **A**) and overall survival (**B**) in first- and second-line metastatic castration-resistant prostate cancer (mCRPC) patients stratified according to 177-lutetium prostate-specific membrane antigen (Lu-PSMA) therapy vs. androgen receptor pathway inhibitor (ARPI)

In PFS analyses between Lu-PSMA vs. ARPI mCRPC patients (Fig. 1A), differences were observed with median PFS of 13.3 (confidence interval [CI]: 7.6–25.7) vs. 8.2 (CI: 6.5–10.1) months (hazard ratio [HR]: 0.70, *p* = 0.16). After multivariable adjustment for potential confounders in patient and tumor characteristics in Cox regression models, Lu-PSMA was also independently associated with better PFS, relative to ARPI treatment (HR: 0.24, *p* = 0.045, Table 2A).

**Table 2:** Univariable und multivariable Cox regression models predicting progression-free survival (PFS; A) and overall survival (OS; B) in first- and second-line metastatic castration resistant prostate cancer (mCRPC) according to treatment with 177-Lutetium prostate-specific membrane antigen radioligand therapy (Lu-PSMA) vs. androgen receptor pathway inhibitor (ARPI) or docetaxel chemotherapy

	Univariable			Multivariable		
PFS	**HR**	**CI**	***p* value**	**HR**	**CI**	***p* value**
ARPI	**Ref**	**-**	**-**	**Ref**	**-**	**-**
Lu-PSMA*	0.70	0.43–1.14	0.16	0.24	0.06–0.97	0.045
Docetaxel	**Ref**	**-**	**-**	**Ref**	**-**	**-**
Lu-PSMA*	0.48	0.28–0.82	< 0.01	0.86	0.18–4.00	0.8
OS	**HR**	**CI**	***p* value**	**HR**	**CI**	***p* value**
ARPI	**Ref**	**-**	**-**	**Ref**	**-**	**-**
Lu-PSMA^+^	0.64	0.32–1.30	0.22	0.065	0.01–0.62	0.01
Docetaxel	**Ref**	**-**	**-**	**Ref**	**-**	**-**
Lu-PSMA^+^	0.34	0.17–0.72	< 0.01	0.03	0.01–0.30	< 0.01

Abbreviation: *HR,* Hazard Ratio; *CI,* Confidence interval; *ECOG,* Eastern Cooperative Oncology Group

*Age at metastatic disease, Gleason Score, ECOG status, metastatic sides at mCRPC, year of treatment

^+^Age at metastatic disease, Gleason Score, ECOG status, metastatic sides at mCRPC, number of systemic treatments for mCRPC, year of treatment

In OS analyses (Fig. 1B), median OS was 68.9 (CI: 41—not reached [NR]) vs. 39.1 (CI: 32.8–63.0) months for Lu-PSMA vs. ARPI, without reaching significance (HR: 0.64, *p* = 0.2). However, after multivariable adjustment, Lu-PSMA was associated with better survival, relative to ARPI treatment (HR: 0.065, *p* = 0.01, Table 2B).

### Baseline characteristics: Lu-PSMA vs. docetaxel

Of all included 269 patients, 11% (*n* = 29) received Lu-PSMA therapy in first- or second-line mCRPC vs. 33% (*n* = 88) docetaxel treatment. In comparison between both treatments (Table 1B), Lu-PSMA patients were significantly older at mCRPC (76 vs. 69 years) and received less systematic treatments for mCRPC in median (2 vs. 3, both *p* < 0.001). Moreover, rates of ECOG 1–2 mCRPC patients were significantly higher in Lu-PSMA than docetaxel patients (76% vs. 41%, *p* < 0.01). Despite not reaching significance, PSA at mCRPC was higher in the Lu-PSMA cohort (23 vs. 12 ng/ml, *p* = 0.09). Moreover, rate of M1c disease at mCRPC was higher in Lu-PSMA patients (11% vs. 2.4%). No further differences were observed regarding De Novo metastatic disease and local therapies to the prostate.

### PFS and OS analyses: Lu-PSMA vs. docetaxel

**Fig. 2:**
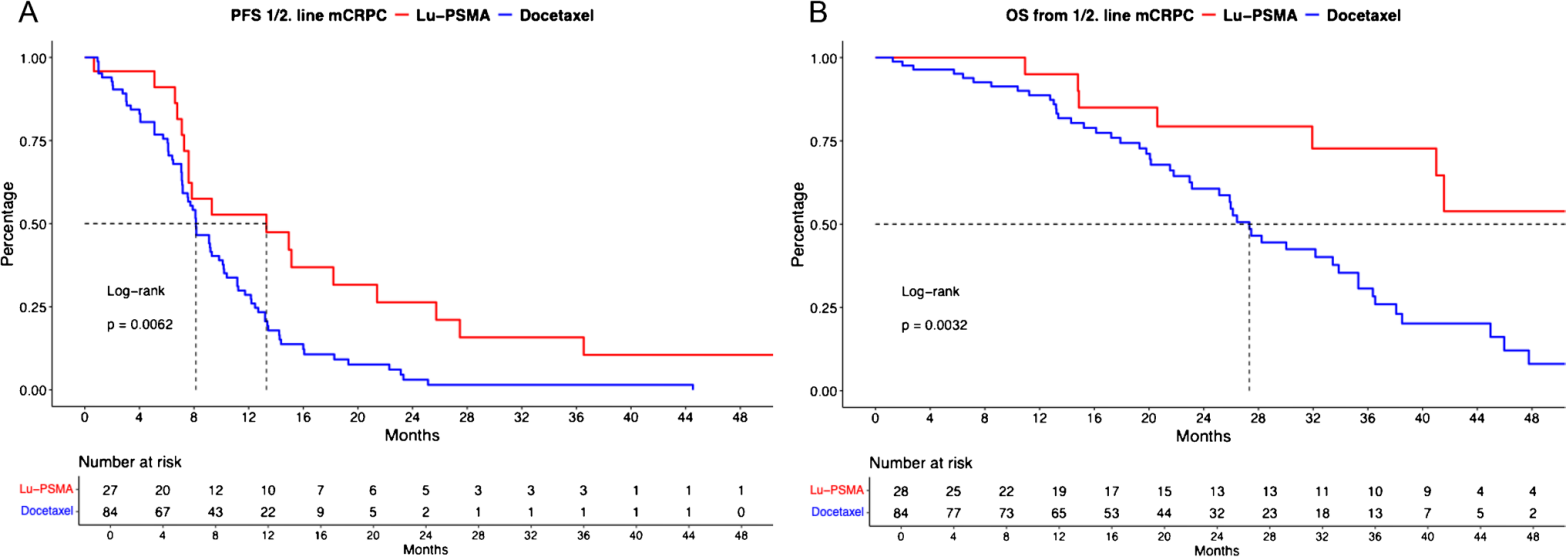
Kaplan Meier curves depicting progression-free survival (PFS, **A**) and overall survival (**B**) in first- and second-line metastatic castration-resistant prostate cancer (mCRPC) patients stratified according to 177-lutetium prostate-specific membrane antigen (Lu-PSMA) therapy vs. docetaxel chemotherapy

In PFS analyses between Lu-PSMA vs. docetaxel mCRPC patients (Fig. 2A), significant differences were observed with median PFS of 13.3 (CI: 7.6–25.7) vs. 8.1 (CI: 7.2–10.1) months (HR: 0.48, *p* < 0.01). After multivariable adjustment for potential confounders in patient and tumor characteristics in Cox regression models, no differences between both treatments were observed (HR: 0.86, *p* = 0.8, Table 2A).

In OS analyses (Fig. 2B), median OS also differed significantly between Lu-PSMA vs. docetaxel chemotherapy with median OS of 68.9 (CI: 41.0-NR) vs. 27.3 (CI: 23.1–35.3) months (HR: 0.34, *p* < 0.01). Moreover, after multivariable adjustment in Cox regression models, Lu-PSMA was significantly associated with better survival, relative to docetaxel treatment (HR: 0.03, *p* < 0.01, Table 2B).

## Discussion

We conducted a real-world study to compare cancer-control outcomes of taxan-naïve mCRPC patients treated with Lu-PSMA after previous ARPI treatment. We hypothesized that in a real-world scenario important differences in cancer-control outcomes may exist regarding PFS and OS outcomes compared to other standard of care treatments. We tested these hypotheses in comparisons for Lu-PSMA vs. ARPI and Lu-PSMA vs. docetaxel and made several important observations.

First, comparing early line mCRPC cohorts with Lu-PSMA treatment, the current study shares similarities to previous made findings, such as high proportions of ECOG status ≥ 2 or advanced median age [14]. Moreover, in specific comparison between Lu-PSMA and ARPI in taxan-naïve mCRPC patients similarities regarding baseline patient and tumor characteristics exist. Despite non-significant higher rates of ECOG status 1–2 (76% vs. 55%) and M1c stage at mCRPC (11% vs. 6%) and significant lower number of received systemic treatments for mCRPC in Lu-PSMA patients (2 vs. 3), no further clinically meaningful or statistically significant differences in baseline characteristics were observed. These findings are not surprising, since ARPI and Lu-PSMA treatments are usually well tolerated and therefore both considered treatment options for either or especially elderly, frail mCRPC patients or those with severe comorbidities [16, 17]. Conversely, when Lu-PSMA taxan-naïve mCRPC patients were compared to docetaxel patients, docetaxel patients were significantly younger in median at mCRPC (seven years), had substantially lower rates of ECOG status 1–2 (39% vs.76%), received less systemic treatment and M1c rate was substantially lower (2.4% vs. 11%). These observations are also in consistence with previous findings. For example, a recently published real-world cohort of elderly mHSPC and mCRPC patients could also demonstrate that with increasing age, fewer sequential treatments are administered and chemotherapy is significantly less frequently used in favor of higher Lu-PSMA treatment rates [18]. All of the above findings further emphasize that Lu-PSMA in a real-world setting is equivalently used in similar patient cohorts as ARPI treatment, while mCRPC patients receiving docetaxel show substantially different baseline characteristics. Still, when comparing tumor burden and location (e.g. M1a vs. M1b vs. M1c) between the three groups, one has to keep in mind that staging was done with PSMA-PET scan for patients prior to Lu-PSMA. This might lead to a higher tumor burden and more patients with M1c in the Lu-PSMA group and might significantly influence especially the results of the multivariable Cox regression analyses.

Second, when cancer-control outcomes between Lu-PSMA vs. ARPI treated taxan-naïve mCRPC patients were compared, also important observations were made. For example, PFS was significantly longer with a five months advantage for Lu-PSMA vs. ARPI treatment (13.3 vs. 8.2 months). Moreover, after additional adjustment for potential confounders in Cox regression models, this advantage was further confirmed. In univariable OS analyses, the benefit of Lu-PSMA failed to reach statistical significance (HR: 0.64, *p* = 0.2), however, a clinically meaningful median OS difference of 68.9 (CI: 41-NR]) vs. 39.1 (CI: 32.8–63.0) months in favor of Lu-PSMA treatment was observed. This advantage is emphasized by the significant survival advantage for Lu-PSMA treatment in multivariable Cox regression models. To the best of our knowledge, no previous real-world evidence conducted a similar head-to-head comparison. However, in the prospective randomized phase III PSMAfore trial, PFS was also significantly better for Lu-PSMA vs. ARPI in taxan-naïve mCRPC patients after previous ARPI treatment (12.0 vs. 5.6 months, HR: 0.42) [11]. However, after a second interim analysis with 45% deaths of the needed deaths according to the power analysis, no significant OS differences was observed, despite a trend towards favoring Lu-PSMA treatment (HR: 0.80, confidence interval 0.48–1.33). Similar to PSMAfore, results of the SPLASH study presented at ESMO 2024 also showed a significantly better PFS for Lu-PSMA, relative to ARPI (9.5 vs. 6.0 months, HR: 0.71) [13]. One explanation for better PFS for Lu-PSMA vs. ARPI treatment might be the suboptimal competitor in the PSMAfore and SPLASH studies and within our real-world study comparison (second-line ARPI), since some previous studies suggest lower PFS rates for ARPI after initial ARPI treatment than with an “change mode of action” [7, 19–21]. Moreover, in both studies – PSMAfore and SPLASH—patients were allowed to cross over to Lu-PSMA (crossover rate of above 84%), which certainly lowers a potential OS benefit compared to our data, where none of the patients from the “comparison-arm” underwent Lu-PSMA therapy as cross-over. However, in our data non-significant differences in OS between both treatments may be explained by different sequential therapies and significantly more systemic treatments for ARPI patients, equalizing the PFS benefit for Lu-PSMA in later therapy lines. This assumption is supported by the fact that Lu-PSMA was superior to ARPI in multivariable adjusted OS models, when for this effect was accounted for. However, these HRs need to be interpreted with caution and may be overfitted due to adjusting for substantial differences in M1c disease at mCRPC also due to different staging modalities, as mentioned above. Moreover, as patients need to have PSMA-positive lesions prior to Lu-PSMA therapy, a certain selection bias might be present, as patients with PSMA-negative lesions (which might be a prognostically unfavorable parameter) are excluded from Lu-PSMA therapy. For example, in the TheraP trial, 27% of patients were excluded due to PSMA-negative lesions or inconclusive lesions within different staging modalities [22].

Finally, when comparison between Lu-PSMA vs. docetaxel taxan-naïve mCRPC patients with previous ARPI treatment was made, also important findings were made. Lu-PSMA was significantly superior in terms of PFS, as well as OS. This is especially of note since Lu-PSMA patients were generally at higher risk of death of any cause due to substantially older median age and advanced frailty status. However, these differences in baseline tumor and patient characteristics are vanished in adjusted multivariable Cox regression models, when docetaxel patients were adjusted to these unfavorable characteristics. One has to keep in mind, that especially PFS might be biased when comparing Lu-PSMA with chemotherapy, because “treatment beyond progression” might be more frequent in patients undergoing Lu-PSMA therapy. A previously published phase II trial also compared Lu-PSMA vs. docetaxel in taxan-naïve patients [23]. Here the authors found significant differences in PFS but not regarding OS when Lu-PSMA vs. docetaxel was compared. However, when comparisons to this trial are made, it must be emphasized that it was designed as a non-inferior trial with the intent to demonstrate similar effects for docetaxel vs. Lu-PSMA. Moreover, within this trial, previous ARPI treatment rates were 60% respectively 70% for Lu-PSMA vs. docetaxel patients. Therefore, inclusion criteria significantly differ compared to our real-world study with this specific inclusion criteria, since standard of care treatment for mHSPC or mCRPC is ARPI [24–27]. With respect to this current standard of care, as well as to approximate inclusion criteria of the PSMAfore study, we specifically chose these patient criteria for comparison.

Our study has limitations which should be acknowledged in its interpretation. Despite the retrospective, single-center design and limitations in sample size of Lu-PSMA-treated patients, further potentially unknown confounders may have influenced outcomes, such as missing data for some variables. Nevertheless, all patients were discussed within a multidisciplinary tumor board, decision to undergo Lu-PSMA therapy was also due to patients’ preference and therefore might lead to a potential selection bias. Moreover, differences in the regime of used Lu-PSMA and applied doses may have occurred. Necessary staging with PSMA-PET/CT prior to Lu-PSMA therapy may resulted in different metastatic sites proportions relative to ARPI/docetaxel treatment and may have substantially influenced multivariable adjusted models. Due to previous PSMA-PET/CT screening patients without PSMA expression did not qualify for Lu-PSMA therapy, which may have introduced a selection bias. Standardized used staging modality in ARPI or docetaxel-treated patients were unfortunately not given, however, reflect real-world clinical scenario. Progression was defined as the beginning of another systemic treatment line which may differ for ARPI/chemotherapy and 177-Lu-PSMA treatment regimens and should be acknowledged in the comparison to trial-derived findings. However, this limitation is shared by similar conducted Lu-PSMA real-world studies [28]. Finally, no information regarding side effects were available.

Taken together, given the limitations of a retrospective study with a potential selection bias, this single-center study including a substantial proportion of patients with treatment preference for Lu-PSMA in taxan-naïve mCRPC setting suggests Lu-PSMA therapy after previous ARPI treatment is associated with more favorable cancer-control outcomes compared to docetaxel and slightly better outcomes relative to ARPI treatment and may therefore be considered as an early treatment option in mCRPC.

## Supplementary Information

Below is the link to the electronic supplementary material.

Supplementary file1 (DOCX 15 KB)

## Data Availability

The datasets generated during and/or analyzed during the current study are available from the corresponding author on reasonable request.

## References

[1] Scher HI, Fizazi K, Saad F, et al. Increased survival with enzalutamide in prostate cancer after chemotherapy. N Engl J Med. 2012;367(13):1187–97. 10.1056/NEJMoa1207506. 22894553 10.1056/NEJMoa1207506

[2] Ryan CJ, Smith MR, de Bono JS, et al. Abiraterone in metastatic prostate cancer without previous chemotherapy. N Engl J Med. 2013;368(2):138–48. 10.1056/NEJMoa1209096. 23228172 10.1056/NEJMoa1209096 PMC3683570

[3] Tannock IF, de Wit R, Berry WR, et al. Docetaxel plus prednisone or mitoxantrone plus prednisone for advanced prostate cancer. N Engl J Med. 2004;351(15):1502–12. 10.1056/NEJMoa040720. 15470213 10.1056/NEJMoa040720

[4] de Bono JS, Oudard S, Ozguroglu M, et al. Prednisone plus cabazitaxel or mitoxantrone for metastatic castration-resistant prostate cancer progressing after docetaxel treatment: a randomised open-label trial. Lancet Lond Engl. 2010;376(9747):1147–54. 10.1016/S0140-6736(10)61389-X. 10.1016/S0140-6736(10)61389-X 20888992

[5] Hussain M, Mateo J, Fizazi K, et al. Survival with olaparib in metastatic castration-resistant prostate cancer. N Engl J Med. 2020;383(24):2345–57. 10.1056/NEJMoa2022485. 32955174 10.1056/NEJMoa2022485

[6] Parker C, Nilsson S, Heinrich D, et al. Alpha emitter radium-223 and survival in metastatic prostate cancer. N Engl J Med. 2013;369(3):213–23. 10.1056/NEJMoa1213755. 23863050 10.1056/NEJMoa1213755

[7] de Wit R, de Bono J, Sternberg CN, et al. Cabazitaxel versus abiraterone or enzalutamide in metastatic prostate cancer. N Engl J Med. 2019;381(26):2506–18. 10.1056/NEJMoa1911206. 31566937 10.1056/NEJMoa1911206

[8] Wenzel M, Siech C, Hoeh B, et al. Contemporary treatment patterns and oncological outcomes of metastatic hormone-sensitive prostate cancer and first- to sixth- line metastatic castration-resistant prostate cancer patients. Eur Urol Open Sci. 2024;66:46–54. 10.1016/j.euros.2024.06.010. 39036044 10.1016/j.euros.2024.06.010 PMC11260326

[9] Wenzel M, Borkowetz A, Lieb V, et al. Efficacy of cabazitaxel in fourth or later line of therapy in metastatic castration-resistant prostate cancer: multi-institutional real-world experience in Germany. Urol Oncol. 2022;40(12):538.e7-538.e14. 10.1016/j.urolonc.2022.09.011. 36244915 10.1016/j.urolonc.2022.09.011

[10] Sartor O, de Bono J, Chi KN, et al. Lutetium-177-PSMA-617 for metastatic castration-resistant prostate cancer. N Engl J Med. 2021;385(12):1091–103. 10.1056/NEJMoa2107322. 34161051 10.1056/NEJMoa2107322 PMC8446332

[11] Morris MJ, Castellano D, Herrmann K, et al. 177Lu-PSMA-617 versus a change of androgen receptor pathway inhibitor therapy for taxane-naive patients with progressive metastatic castration-resistant prostate cancer (PSMAfore): a phase 3, randomised, controlled trial. Lancet Lond Engl. 2024;404(10459):1227–39. 10.1016/S0140-6736(24)01653-2. 10.1016/S0140-6736(24)01653-2 PMC12121614 39293462

[12] Emmett L, Subramaniam S, Crumbaker M, et al. [177Lu]Lu-PSMA-617 plus enzalutamide in patients with metastatic castration-resistant prostate cancer (ENZA-p): an open-label, multicentre, randomised, phase 2 trial. Lancet Oncol. 2024;25(5):563–71. 10.1016/S1470-2045(24)00135-9. 38621400 10.1016/S1470-2045(24)00135-9

[13] Sartor O, Jiang DM, Smoragiewicz M et al. Efficacy of 177Lu-PNT2002 in PSMA-positive mCRPC following progression on an androgen-receptor pathway inhibitor (ARPI) (SPLASH). Ann Oncol. 2024;35(suppl_2): 1–72. 10.1016/annonc/annonc1623.

[14] Satapathy S, Yadav MP, Ballal S, Sahoo RK, Bal C. [177Lu]Lu-PSMA-617 as first-line systemic therapy in patients with metastatic castration-resistant prostate cancer: a real-world study. Eur J Nucl Med Mol Imaging. 2024;51(8):2495–503. 10.1007/s00259-024-06677-y. 38467922 10.1007/s00259-024-06677-y

[15] Mader N, Nguyen Ngoc C, Kirkgöze B, et al. Extended therapy with [177Lu]Lu-PSMA-617 in responding patients with high-volume metastatic castration-resistant prostate cancer. Eur J Nucl Med Mol Imaging. 2023;50(6):1811–21. 10.1007/s00259-023-06119-1. 36702927 10.1007/s00259-023-06119-1 PMC10119067

[16] Heck MM, Tauber R, Schwaiger S, et al. Treatment outcome, toxicity, and predictive factors for radioligand therapy with 177Lu-PSMA-I&T in metastatic castration-resistant prostate cancer. Eur Urol. 2019;75(6):920–6. 10.1016/j.eururo.2018.11.016. 30473431 10.1016/j.eururo.2018.11.016

[17] Cao B, Kim M, Reizine NM, Moreira DM. Adverse events and androgen receptor signaling inhibitors in the treatment of prostate cancer: a systematic review and multivariate network meta-analysis. Eur Urol Oncol. 2023;6(3):237–50. 10.1016/j.euo.2023.01.001. 36682938 10.1016/j.euo.2023.01.001

[18] Fernando M, Anton A, Weickhardt A, et al. Treatment patterns and outcomes in older adults with castration-resistant prostate cancer: analysis of an Australian real-world cohort. J Geriatr Oncol. 2023;14(8): 101621. 10.1016/j.jgo.2023.101621. 37683368 10.1016/j.jgo.2023.101621

[19] Yamada Y, Matsubara N, Tabata KI, et al. Abiraterone acetate after progression with enzalutamide in chemotherapy-naïve patients with metastatic castration-resistant prostate cancer: a multi-center retrospective analysis. BMC Res Notes. 2016;9(1):471. 10.1186/s13104-016-2279-9. 27756383 10.1186/s13104-016-2279-9 PMC5069876

[20] Suzuki H, Castellano D, de Bono J, et al. Cabazitaxel versus abiraterone or enzalutamide in metastatic castration-resistant prostate cancer: post hoc analysis of the CARD study excluding chemohormonal therapy for castrate-naive disease. Jpn J Clin Oncol. 2021;51(8):1287–97. 10.1093/jjco/hyab028. 33738495 10.1093/jjco/hyab028 PMC8521736

[21] Broyelle A, Delanoy N, Bimbai AM, et al. Taxanes versus androgen receptor therapy as second-line treatment for castrate-resistant metastatic prostate cancer after first-line androgen receptor therapy. Clin Genitourin Cancer. 2023;21(3):349-356.e2. 10.1016/j.clgc.2023.02.006. 36935298 10.1016/j.clgc.2023.02.006

[22] Hofman MS, Emmett L, Sandhu S, et al. Overall survival with [177Lu]Lu-PSMA-617 versus cabazitaxel in metastatic castration-resistant prostate cancer (TheraP): secondary outcomes of a randomised, open-label, phase 2 trial. Lancet Oncol. 2024;25(1):99–107. 10.1016/S1470-2045(23)00529-6. 38043558 10.1016/S1470-2045(23)00529-6

[23] Satapathy S, Mittal BR, Sood A, et al. [177Lu]Lu-PSMA-617 versus docetaxel in chemotherapy-naïve metastatic castration-resistant prostate cancer: final survival analysis of a phase 2 randomized, controlled trial. J Nucl Med Off Publ Soc Nucl Med. 2023;64(11):1726–9. 10.2967/jnumed.123.266141. 10.2967/jnumed.123.266141 37709534

[24] Cornford P, Tilki D, van den Bergh RCN et al. EAU Guidelines on Prostate Cancer. Edn. presented at the EAU Annual Congress Paris 2024. ISBN 978-94-92671-23-3.

[25] Hoeh B, Garcia CC, Wenzel M, et al. Triplet or doublet therapy in metastatic hormone-sensitive prostate cancer: updated network meta-analysis stratified by disease volume. Eur Urol Focus. Published online April 11, 2023:S2405-4569(23)00094-9. 10.1016/j.euf.2023.03.024. 10.1016/j.euf.2023.03.024 37055323

[26] Mandel P, Hoeh B, Wenzel M, et al. Triplet or doublet therapy in metastatic hormone-sensitive prostate cancer patients: a systematic review and network meta-analysis. Eur Urol Focus. Published online September 1, 2022:S2405-4569(22)00176-6. 10.1016/j.euf.2022.08.007. 10.1016/j.euf.2022.08.007 36058809

[27] Wenzel M, Würnschimmel C, Nocera L, et al. Overall survival after systemic treatment in high-volume versus low-volume metastatic hormone-sensitive prostate cancer: systematic review and network meta-analysis. Eur Urol Focus. Published online April 11, 2021:S2405-4569(21)00109–7. 10.1016/j.euf.2021.04.003. 10.1016/j.euf.2021.04.003 33853754

[28] Wenzel M, Koll F, Hoeh B et al. Real-world comparison of cabazitaxel versus ^177^Lu-PSMA radiopharmaceutical therapy in metastatic castration-resistant prostate cancer. J Nucl Med. 2024 Nov 14:jnumed.124.268807. 10.2967/jnumed.124.268807. 10.2967/jnumed.124.268807 39542702

