# Supplementary material for: Lutetium-177 PSMA radioligand therapy in taxan-naive first- and second-line metastatic castration resistant prostate cancer after first-line ARPI therapy
Source: Eur J Nucl Med Mol Imaging. 2025 Jan 13;52(6):2015–22. doi: 10.1007/s00259-025-07076-7 (PMC12014794; doi:10.1007/s00259-025-07076-7)
Supplement: Supplementary file 1 — Supplementary file1 (DOCX 15 KB) [file 259_2025_7076_MOESM1_ESM.docx]

For PET/CT imaging [^68^Ga]Ga-PSMA-11 was administered intravenously, aiming for a target activity of 2.0 MBq/kg body weight. ^68^Gallium was obtained from a ^68^Ge/^68^Ga radionuclide generator (GalliaPharm, Eckert & Ziegler Radiopharma, Berlin, Germany). Scans were performed on a Biograph 6 PET/CT scanner (Siemens, Erlangen, Germany) applying decay, scatter and attenuation correction in accordance with the procedure guidelines set out by the joint EANM and SNMMI consensus statement [1]. Whole-body images (vertex to mid-thigh) were acquired 60 minutes after tracer injection. PET acquisition time was 4 minutes per bed position. CT data was used for attenuation correction and anatomical correlation, the X-ray tube voltage being set to 130 kV with a modulated tube current (CARE Dose 4D, Siemens, Erlangen, Germany).

[^177^Lu]Lu-PSMA-617 (12 patients) or [^177^Lu]Lu-PSMA-I&T (16 patients) was given by intravenous injection over 30-60 s and concomittant administration of saline solution (1000 mL). In accordance with radioprotection regulations, radioligand therapy was performed as an in-patient procedure at the nuclear medicine therapy ward. Patients recieved a median of 3 (IQR 2-6) cycles in 4–6-week intervals. The median activity per cycle was 7.4 (IQR 6.4-8.5) GBq, cumulative activity was 22.2 (IQR 14.9-44.8) GBq (data on radiopharmaceutical activity was unavailable for one patient). The administered activities could be modified in patients with potential risks for renal or hematological toxicity.

1. Fendler, W.P.; Eiber, M.; Beheshti, M.; Bomanji, J.; Ceci, F.; Cho, S.; Giesel, F.; Haberkorn, U.; Hope, T.A.; Kopka, K.; et al. 68Ga-PSMA PET/CT: Joint EANM and SNMMI Procedure Guideline for Prostate Cancer Imaging: Version 1.0. Eur J Nucl Med Mol I 2017, 44, 1014–1024, doi:10.1007/s00259-017-3670-z.
